# Toys in the Playrooms of Children’s Hospitals: A Potential Source of Nosocomial Bacterial Infections?

**DOI:** 10.3390/children8100914

**Published:** 2021-10-14

**Authors:** Viktorija Aleksejeva, Anastasija Dovbenko, Juta Kroiča, Ingus Skadiņš

**Affiliations:** 1 Department of Residency, Rīga Stradiņš University, LV-1007 Riga, Latvia; va.aleksejeva@gmail.com; 2 Department of Biology and Microbiology, Rīga Stradiņš University, LV-1007 Riga, Latvia; anastasija.dovbenko@rsu.lv (A.D.); juta.kroica@rsu.lv (J.K.)

**Keywords:** toys as vector, nosocomial infection, bacteria on toys, pediatrics

## Abstract

Pediatric patients are more susceptible and vulnerable to nosocomial infections, in part because of their nascent and developing immune system and in part due to certain congenital conditions. Consequently, we found limited literature that investigated and reported children’s toys in hospital playrooms as potential reservoirs of pathogenic microbes. Hence, in the present study, we aimed to investigate toys as potential vectors for nosocomial infections in children’s hospitals. Microbiological samples from 120 toys were collected between April 2018 and November 2018. The specimens were cultivated on suitable cultivation agars for 24–72 h at 37 °C and CFU/cm^2^ (colony forming units) was determined. Antibiotic susceptibility testing was performed using disc diffusion and E-tests. Our results indicate that 84% of samples were contaminated with different microbes. Four distinct genera and thirty-seven species of bacteria were identified. The most frequently isolated pathogen was *Sphingomonas paucimobilis* (>603 CFU/cm^2^). Most of the identified microorganisms were members of normal human microbiota. Although *Staphylococcus aureus* and *Acinetobacter baumannii* were identified, CFU/cm^2^ was relatively low and they were found to be sensitive to antibiotics. Additionally, plastic toys showed the highest average CFU/cm^2^ of 91.9. Our results bolster the need for adoption and strict enforcement of proper disinfection techniques for toys in the hospital playrooms.

## 1. Introduction

Hospital-acquired infections or nosocomial infections are infections that are acquired in a healthcare institution/setting that are not the primary reason for hospitalization [1]. Pediatric patients in general are more susceptible to these types of infection due to a variety of reasons including a developing immune system, certain congenital conditions, and other factors that increase the risk of hospital-acquired infections [2,3,4]. Nosocomial infections in children pose a significant challenge, especially for the physicians, as they are typically associated with increased mortality and higher treatment costs [3,5].

Multiple factors that are associated with an increase in the risk of acquiring nosocomial infections in pediatric hospitals are reported in the literature. In a study by Washman et al., it was shown that reducing the number of visitors decreased the number of hospital-acquired acute respiratory viral infections in non-intensive care units from 3.37 to 2.14 cases per 1000 risk patients a day [6], thereby highlighting the number of visitors as an important and often underscored factor that can significantly affect the incidence and spread of hospital-acquired infections in the pediatric units [4]. In separate research by Urrea et al., the authors showed that the patients with a central venous line and/or patients receiving parenteral nutrition were at a higher risk of developing hospital-acquired infections [2]. Central venous lines are commonly used in the pediatric practice, thereby amplifying the associated risk of acquiring nosocomial infections in the pediatric population [7]. Data reveals that the surroundings (environment) also have an effect on the spread of these infections. For example, Pillet et al., found that specific viruses (enteric and respiratory viruses) were present on the mobile phones of the healthcare staff in the pediatric units more often when compared to the staff in other units [8].

Information regarding toys found in the playrooms at pediatric hospitals is very limited. The playrooms at the hospitals serve to distract children and relieve stress that is associated with sickness and hospitalization. Playing also stimulates a therapeutic effect on hospitalized children, thereby improving their physical and emotional wellbeing [9]. Furthermore, the physicians use toys as an aid in the examination of the children [9]. Although toys could be considered as a positive addition to the hospital environment, they could also play a critical role in the spread of hospital-acquired infections. Children often tend to disregard established and posted hygienic precautions (both intentionally and unintentionally) while playing, insert fingers into their mouth and nose and occasionally do not wash their hands prior to eating or after using the toilet, which leads to the contamination of the surroundings with microorganisms, including harmful pathogens. Currently, results of any quality research that could confirm or deny these assumptions and analyze the safety of these toys from the perspective of the hospital-acquired infections are lacking in literature. Hence, the aim of this research was to determine the diversity and the count of microorganisms on different toys in different units at the children’s hospital and consequently assess the risk of these toys being a source of hospital-acquired infections.

## 2. Materials and Methods

### 2.1. Sampling and Identification of Bacteria

For bacterial identification, samples from 120 toys were collected from April 2018 to November 2018. Each sample was taken from a specified area (2 × 2 cm) of each toy using sterile swabs that were priorly moistened in sterile 0.9% saline. Specimens were collected from toys of several playrooms of the Children’s Clinical University Hospital (CCUH), Riga, Latvia, located in the: (1) cardiology, cardiac surgery, surgery department, (2) gastroenterology, rheumatology, endocrinology department, (3) lobby, (4) day-case unit, (5) neurology, neurosurgery, nephrology department, (6) ophthalmology, otorhinolaryngology department, (7) oncology department, and (8) the multidisciplinary department.

The patient demographics of these departments encompassed children younger than 18 years old accompanied by one of their adult guardians, in most cases. During our research period, the sanitation recommendations and cleaning schedule for CCUH playroom toys was followed. The typical regimen consisted of hand-cleaning and sanitizing toys at least two time per day.

Specimen swabs were inserted into the test tubes containing two milliliters of sterile saline (0.9%) and subsequently transported to the microbiology laboratory at Rīga Stradiņš University (RSU), Riga, Latvia. Test tubes with the swabs (2 mL) were further sonicated for one minute at 40 kHz (Ultrasonic cleaner 08855-02, Cole-Parmer, Chicago, Illinois, USA). Post-sonication, the samples were vortexed and inoculated on blood agar (TSA, Oxoid™, Basingstoke, United Kingdom) plus 6% defibrinated sheep blood, Levine EMB agar (Oxoid™, Basingstoke, United Kingdom) and mannitol salt agar (Oxoid™, Basingstoke, United Kingdom). Samples were cultivated for 24–72 h at 37 °C. After incubation, the colony forming units (CFU) were counted on the plates and determined as CFU/cm^2^. Additionally, CFU/toy was determined. The colonies were stained using Gram staining method and evaluated using a light microscope (Nikon Eclipse E200, Tokyo, Japan). Microorganisms were identified using the automated VITEK2 method (Compact 30 bioMérieux, Marcy-l’Étoile, France).

### 2.2. Antibacterial Susceptibility Testing 

Quantitative antibacterial susceptibility testing was performed using the E-test (Epsilometer test) whilst standard disc diffusion method was employed as a qualitative test. The E-test (Liofilmchem^®^ s.r.l., Roseto degli Abruzzi, Italy) for vancomycin with determination of MIC (minimum inhibitory concentration) was performed in the cases where *Sphingomonas paucimobilis* and *Enterococcus casseliflavus* cultures were identified. Bacterial suspension for antibacterial susceptibility testing was prepared according to the manufacturer’s recommendations with an optical density of 0.5 McFarland. Cultivation was performed using the Mueller–Hinton solid medium (Biolife, Milan, Italy).

In case of *Acinetobacter baumanii* specimens, the Kirby-Bauer disc diffusion method was used for the following antibiotics using the CLSI (Clinical and Laboratory Standards Institute) (Annapolis Junction, Maryland, USA) guidelines: doripenem (10 µg), meropenem (10 µg), levofloxacin (5 µg), gentamicin (10 µg), trimethoprim-sulfamethoxazole (25 µg (1.25/23.75)), tobramycin (10 µg), ciprofloxacin (5 µg) (Liofilmchem^®^ s.r.l., Roseto degli Abruzzi, Italy). In the case of *S. aureus* specimens, the Kirby–Bauer disc diffusion method was used for the following antibiotics: linezolid (10 µg), cefoxitin (30 µg), levofloxacin (5 µg), netilmicin (30 µg), tetracycline (30 µg), erythromycin (15 µg), gentamicin (10 µg), rifampicin (5 µg) (Liofilmchem^®^ s.r.l., Roseto degli Abruzzi, Italy).

## 3. Results

### 3.1. Results of the Identification of Bacteria

The samples were collected from a total of 120 different toys from April 2018 to November 2018. Out of the 120 samples that were analyzed, samples from 101 toys were found to be positive, i.e., at least one colony forming unit was detected (henceforth referred to as positive culture). Plastic toys showed the highest average CFU/cm^2^ (92), followed by the wooden toys (34), cardboard toys (26), stuffed toys (13), rubber toys (4), and the metal toys (1) (Table 1).

### 3.2. Diversity of Identified Microorganisms in Isolated Samples

**Figure 1:**
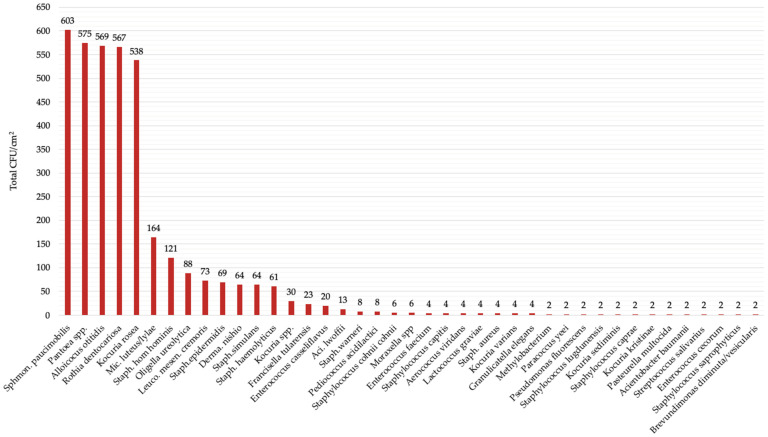
Total CFU/cm^2^ of identified microorganisms.

We found that the most isolated microbe from the toys was *Sphingomonas paucimobilis* (>603 CFU/cm^2^), followed by *Panotea spp.* (>575 CFU/cm^2^), *Alloiococcus otitidis* (>569 CFU/cm^2^), *Rothia dentocariosa* (>567 CFU/cm^2^) and *Kocuria rosea* (>538 CFU/cm^2^) (Figure 1).

A total of 4 genera and 37 species of microorganisms were identified. Since the identified number of microorganisms in the neurology, neurosurgery, nephrology department, the ophthalmology, otorhinolaryngology department, the oncology department and the multidisciplinary department were relatively low (average CFU less than 20) and the diversity of identified microorganism was poor, detailed analysis was only applied to the remaining departments of cardiology, cardiac surgery, surgery, gastroenterology, rheumatology, endocrinology department, the day-case unit and the lobby.

The highest average CFU/cm^2^ (≥65.5) was found in the specimens from the toys in the departments of cardiology, cardiac surgery, and surgery at the Children’s Clinical University Hospital. A total of 20 samples were collected from these departments. Positive cultures were found in 19 samples (95%) of these samples, from which 14 (74%) samples were plastic, followed by four wooden toys (21%) and a single (5%) metal toy. The most common microorganism identified was *Pantoea spp.* (565 CFU/cm^2^), followed by *Sphingomonas paucimobilis* (563 CFU/cm^2^), and *Dermacoccus nishinomiyaensis* (60 CFU/cm^2^).

The average CFU/cm^2^ from the samples collected at the departments of gastroenterology, rheumatology, and endocrinology was ≥35.2. Out of the 18 (90%) samples, at least one colony grew from 14 (78%) plastic and 4 (22%) wooden toys. The most common microorganisms were *Rothia dentocariosa* (563 CFU/cm^2^), *Kocuria rosea* (363 CFU/cm^2^) and *Oligella ureolytica* (88 CFU/cm^2^). The third highest average according to CFU/cm^2^ (≥24.7) was found in the samples from the playroom in the lobby of the Children’s Clinical University Hospital. Positive cultures were retrieved from 18 (90%) samples and all the toys were made of plastic.

**Figure 2:**
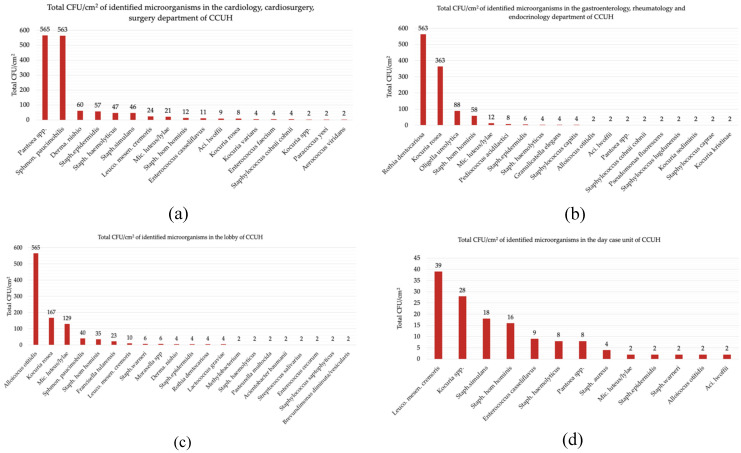
Diversity of identified microorganisms in different departments of CCUH. (**a**) Total CFU/cm^2^ of identified microorganisms in the cardiology, cardiac surgery, surgery department; (**b**) total CFU/cm^2^ of identified microorganisms in the gastroenterology, rheumatology, endocrinology department; (**c**) total CFU/cm^2^ of identified microorganisms in the lobby of CCUH; (**d**) total CFU/cm^2^ of identified microorganisms in the day-case unit.

The average CFU/cm^2^ in samples from the day-case unit was 8.2. All 20 samples had a positive culture with 15 (75%) samples derived from the plastic toys, followed by 3 (15%) stuffed toys and 2 (10%) wooden toys. *Leuconostoc mesenteroides* (39 CFU/cm^2^) was the most common microbe, followed by *Kocuria spp.* (28 CFU/cm^2^) and *Staphylococcus simulans* (18 CFU/cm^2^) (Table 2, Figure 2).

### 3.3. Antibacterial Susceptibility

According to the disc diffusion method (following CLSI guidelines), isolated *S. aureus* samples were susceptible to the following antibiotics: linezolid (10 µg), cefoxitin (30 µg), levofloxacin (5 µg), netilmicin (30 µg), tetracycline (30 µg), erythromycin (15 µg), gentamicin (10 µg), and rifampicin (5 µg) (Liofilmchem^®^ s.r.l., Roseto degli Abruzzi, Italy). An *Acinetobacter baumanii* sample isolated from one toy in the lobby was susceptible to the following antibiotics: doripenem (10 µg), meropenem (10 µg), levofloxacin (5 µg), gentamicin (10 µg), trimethoprim-sulfamethoxazole (25 µg (1.25/23.75)), tobramycin (10 µg), and ciprofloxacin (5 µg) (Liofilmchem^®^ s.r.l., Roseto degli Abruzzi, Italy).

Five pure cultures of *Sphingomonas paucimobilis* and one pure culture of *Enterococcus casseliflavus* were tested using the E-test method (Liofilmchem^®^ s.r.l.,Roseto degli Abruzzi Italy), to determine their susceptibility against vancomycin. *Sphingomonas paucimobilis* had the following MIC values: MIC 0.25 µg/mL, MIC 0.125 µg/mL, MIC 0.19 µg/mL, and MIC 0.064 µg/mL. The results indicate susceptibility to vancomycin (breakpoint MIC 2–3 µg/mL [10]), whereas the MIC of *Enterococcus casseliflavus* was 0.125 µg/mL, also indicating susceptibility to vancomycin (according to EUCAST guidelines).

## 4. Discussion

In the present study we found that the microorganisms can colonize a wide variety of toys. The number of the positive cultures from the collected samples remained high during the study period (101 out of 120 samples, or 84%). Few studies in the past have evaluated toys as a potential source of nosocomial bacterial infections. For example, in a study by Boretti et al., 52 out of 60 toys were reported as positive (87%) for *Staphylococcus spp.* with plastic toys being reported as the most contaminated, which is similar to the results obtained in the present study [11]. Querido et al., have indicated that one of the main reasons for the development of hospital-acquired infections, according to the WHO, is the ability of microorganisms to survive on dry surfaces for a prolonged period of time [12]. Considering the demographic characteristics of the age group analyzed in this research, the high number of positive cultures could be explained by less stringent adherence to hygienic measures with a special emphasis on the hand hygiene, and children’s general tendency to insert fingers in their mouth and nose. These factors increase the risk of the transmission of microorganisms, including those present on toys [4,13], Additionally, when children play with the toys and have not washed their hands after meals or after using the toilet, food particles and patients’ biological material can contaminate the surroundings, including the surface of toys, and thus provide nutrients for microorganisms, promoting their survival in the environment.

Another aspect that must be evaluated when considering toys as a potential source of hospital-acquired infections is their cleaning and maintenance. In research by Hashi et al., it was shown that strict adherence to proper hand hygiene is efficacious in reducing the transmission of bacterial nosocomial infections associated with diarrhea. In one of the groups that included children up to the age of 59 months (4–5 years), a strict hand hygiene regimen was introduced by the authors. This resulted in a significant decrease in the number of cases of diarrheal disease by up to 35% [14]. Therefore, it is important to analyze whether the methods that are currently applied are effective. For example, Ibfelt et al., examined if cleaning and disinfection of toys every two weeks reduced the number of infections in a kindergarten setting. The results revealed that although the number of microorganisms on toys was reduced, the rate of infections remained unchanged [13]. It is important to note that this analysis of contamination of toys was carried out before the COVID-19 pandemic, which means that the circumstances have changed, and during the pandemic, hospitals have augmented their procedures in infection control, including the cleaning and disinfection of toys.

Our research identified a wide spectrum of bacterial species (37 species). Most species were found to be members of the normal microbiota or caused opportunistic bacterial infections. The most common genus was *Sphingomonas*, with *Sphingomonas paucimobilis* being the predominantly isolated bacteria. It is a Gr- opportunistic bacteria that can survive in the hospital environment for extended periods of time with low levels of nutrients [15]. It was most frequently isolated from the playrooms in the departments of cardiology, cardiac surgery, and surgery. Although this microorganism is considered as an opportunistic microorganism with low pathogenicity, several cases of bacteremia in previously healthy children have been reported [16,17]. In a report by Ryan et al., the authors listed multiple cases in which the pathogenic microorganism recovered from patients was *Sphingomonas paucimobilis* and was associated with bacteremia/septicemia, peritonitis, lung infections and other diseases [15]. Meanwhile, in another study by Bayram et al., the authors found that in the 24 patients with a confirmed *Sphingomonas paucimobilis* infection, 11 cases were hospital-acquired infections [17]. In a previous report, three patients had received empirical antibacterial treatment with vancomycin with successful results, which prompted us to perform susceptibility testing against vancomycin in *Sphingomonas paucimobilis* isolated in our research. An MIC breakpoint of 2–3 µg/mL was used based on the research by Angelakis et al., in which the susceptibility of *Sphingomonas mucosissima* to several antibiotics was determined, including vancomycin [10]. Results of our research indicate that all four cultures of *Sphingomonas paucimobilis* that were isolated and analyzed were susceptible to vancomycin.

The second most common genus identified in the present study was *Pantoea* (575 CFU/cm^2^). The members of this genus were mostly isolated from toys in the playrooms of the departments of cardiology, cardiac surgery, and surgery. Some microorganisms of this genus are considered opportunistic, including *Pantoea agglomerans*, which has been shown as a causative agent of bacteremia in cases of contaminated intravenous solutions and parenteral nutrition in the hospital environment [18,19,20]. Several clinical cases have also identified *Pantoea agglomerans* as the causative agent of nosocomial infection in pediatric patients [20,21]. *Alloicoccus otitidis* was also identified in our research with 569 CFU/cm^2^ and has been indicated as a causative agent of otitis media in children [22,23]. *Kocuria rosea* (538 CFU/cm^2^) colonizes the mucosa of the throat and the skin with multiple clinical cases published identifying it as a causative agent for infections, including endocarditis and peritonitis in pediatric population [24,25,26].

Though rarely isolated on the surface of the toys, several microorganisms associated with multi-drug resistance were identified in our study, including *Staphylococcus aureus* and *Acinetobacter baumanii*. Antibacterial susceptibility testing revealed that these microorganisms were susceptible to commonly used antibiotics. Yet, in a research by Boretti et al., the samples collected from the toys found in the pediatric units yielded several multi-drug resistant strains of *S. aureus* (71.4% penicillin, 35.7% clindamycin, 28.6% clarythromycin, 21.4% oxacillin) [11]. Furthermore, in the research by Nzeako et al., samples collected from different surfaces, including children’s toys, showed wide diversity of microorganisms, including *Acinetobacter* spp., which the authors found to be resistant to different antibiotics [27]. Additionally, in the research published by Buttery et al., bath toys were reported as a possible source of multi-drug resistant *Pseudomonas aerugionsa* isolates which are generally associated with nosocomial infection in the pediatric oncology department [28].

Nonetheless, during our study period we faced several limitations that need to be highlighted. First, while some form of sanitation recommendations and cleaning schedules for playroom toys existed before, during, and after the study period, these recommendations were not readily available for us during our research. To learn about these recommendations, we had to conduct separate interviews with the working hospital personnel. Secondly, we lacked information regarding the number of patients that usually visited the playrooms per day. We believe that this additional information would have provided us with a better understanding of our results and would have helped us conclude more accurately as to why the cardiology, cardiac surgery, and surgery department showcased the highest caseloads amongst all the other departments. Lastly, since we did not have information regarding the history of nosocomial infections in CCUH or each of the departments separately during our research, we could not clinically correlate our laboratory findings. Nevertheless, we want to emphasize that the toys remain an important and integral part of the playrooms in the healthcare setting for pediatric patients.

## 5. Conclusions

Toys from the cardiology, cardiac surgery, surgery department playroom showed the highest average CFU/cm^2^. Most of the identified microorganisms were members of the normal microbiota. Microorganisms, such as *S. aureus* and *A. baumannii*, were also identified, however the obtained CFU/cm^2^ were relatively low and all the isolated strains were susceptible to commonly used antibiotics. Microorganisms can survive on the toys in relatively high numbers, which makes it necessary to pay increased attention towards thorough disinfection, as well as towards the material composition and the size of toys in the hospital playrooms to limit the spread of nosocomial infections.

## Figures and Tables

**Table 1:** Average CFU/cm^2^ and the number of positive inoculations in the toys of different materials.

	Average CFU/cm^2^	Toys with Positive Sample	Total Toys	Positive Samples (%)
Plastic toys	92	74	85	87.1%
Wooden toys	34	16	19	84.2%
Cardboard toys	26	1	3	33.1%
Stuffed toys	13	7	9	77.8%
Rubber toys	4	1	1	100.0%
Metal toys	1	2	3	66.7%

**Table 2:** Bacterial colonization of toys in the cardiology, cardiac surgery, surgery department, the gastroenterology, rheumatology, endocrinology department, the lobby and the day-case unit of CCUH.

	Average CFU/cm^2^	Average CFU/Toy	Number of Positive Samples	Positive Samples (%)
Cardiology, cardiac surgery, and surgery department	65.5	38.2	19	95%
Gastroenterology, rheumatology, endocrinology department	35.2	28.5	18	90%
Lobby	24.7	26.8	18	90%
Day-case unit	8.2	3.6	20	100%

## Data Availability

The corresponding datasets of this study are available from the corresponding author on reasonable request.
